# Breathing in harm: current insights into the effects of air pollution on cardiorespiratory health

**DOI:** 10.4103/mgr.MEDGASRES-D-25-00201

**Published:** 2026-09-12

**Authors:** Vasiliki Michou, Dimitrios Tsamos, Aikaterini Itziou, Constantinos Tsanaktsidis

**Affiliations:** 1 Department of Midwifery, School of Healthcare Sciences, University of Western Macedonia, Keptse, Ptolemaida, Greece; 2 Sports Medicine Laboratory, School of Physical Education and Sport Science, Aristotle University, Thessaloniki, Greece; 3 Department of Mechanical Engineering, School of Engineering, University of Western Macedonia, Kozani, Greece; 4 Department of Chemical Engineering, School of Engineering, University of Western Macedonia, Kozani, Greece

**Keywords:** air pollution, air quality standards, cardiometabolic risk, cardiorespiratory health, cardiovascular disease, chronic obstructive pulmonary disease, mitigation strategies, pulmonary disease, vulnerable populations

## Abstract

Air pollution significantly contributes to cardiovascular and respiratory diseases, leading to increased morbidity and mortality. This review critically examines the mechanisms linking air pollution to higher cardiometabolic risk and cardiovascular death. It examines the impact of air pollution on respiratory health, specifically its role in exacerbating conditions such as heart failure, chronic obstructive pulmonary disease, and asthma. Special attention is given to vulnerable groups, including pregnant individuals, neonates, children, and patients with pre-existing cardiovascular or respiratory conditions, who are particularly susceptible and experience more severe health effects. Emerging evidence regarding the interactions between air pollution and coronavirus disease 2019 is also presented, showing how reductions in pollution levels during pandemic lockdowns were associated with improved cardiopulmonary health. Furthermore, the review discusses international differences in air quality standards and recent updates to exposure limits for air pollutants, as well as their implications for public health. It evaluates various mitigation strategies, including policy interventions, technological solutions, and approaches to reduce risk-based exposure. Lastly, the review emphasizes the need for comprehensive, evidence-based frameworks to minimize exposure and prevent diseases associated with pollution. Strengthening air quality management is essential for reducing the global burden of cardiovascular and respiratory diseases.

## Introduction

Climate significantly affects air quality, with factors such as pollutant emissions, transportation, and dispersion influenced by temperature, humidity, wind patterns, and vertical mixing.^1^ Climate change is expected to worsen air quality in densely populated areas by altering atmospheric circulation, precipitation patterns, and chemical processes.^2^ Air pollution is defined as the presence of chemical and biological substances in the atmosphere that may pose risks to human health and the environment.^3^ Based on that, air pollution consists of a complex and dynamic mixture of PM, gases and gaseous co-pollutants, whose composition and sources can vary significantly across locations and over time.^3^,^4^ The major conventional air pollutants used to quantify exposure firstly include particulate matters (PMs), which are classified into coarse (PM_10_, mean aerodynamic diameter 10–2.5 μm), fine (PM_2.5_, 2.5–0.1 μm), and ultrafine (PM_0.1_, < 0.1 µm) particles based on size.^5^ Gaseous pollutants include nitrogen monoxide (NO), nitrogen dioxide (NO_2_), sulfur monoxide (SO), sulfur dioxide (SO_2_) and carbon monoxide (CO), as well as volatile organic compounds such as benzene, acrolein, and butadiene. PM_10_ is typically generated from mechanical activities like industrial processes, mining, and construction, whereas PM_2.5_ mainly results from the combustion of fossil fuels, biomass, and crop residue burning. Ultrafine particles and volatile organic compounds are largely attributed to fresh emissions from motor vehicles.^6^ Motor vehicles and industrial facilities are also major sources of gaseous air pollutants, particularly nitrogen oxides (NO_x_), which are primarily generated through the high-temperature combustion of fossil fuels. These processes occur in engines, power plants, and manufacturing operations, where elevated temperatures facilitate the reaction between atmospheric nitrogen and oxygen, resulting in the formation of NO and NO_2_.^7^ While SO_2_ primarily originates from fossil fuel combustion, particularly from automobile exhaust.^8^ Ozone (O_3_) is the most common secondary pollutant, formed through photochemical reactions, and exists in both gas and particle phases along with various inorganic and organic acids.^9^

Increasingly, climate change and air pollution are recognized as deeply interconnected crises that together are driving a substantial rise in the global burden of disease.^10^,^11^ Poor air quality not only threatens ecosystem stability but also significantly impacts public health, contributing to a wide range of acute and chronic health conditions, especially cardiovascular and respiratory diseases. As global temperatures rise and pollutant levels intensify, urgent and coordinated action is needed to mitigate both climate change and its compounding effects on air pollution and human health.^2^ Air pollution currently affects nearly the entire global population, with the World Health Organization (WHO) reporting in 2019 that 99% of people breathe air exceeding guideline limits and containing harmful levels of pollutants, with the greatest exposures occurring in low- and middle-income countries.^12^ It reduces average life expectancy by nearly 3 years, more than acquired immunodeficiency syndrome smoking and is ranked as the 9th most significant modifiable risk factor for disease, surpassing physical inactivity, high cholesterol, and substance abuse.^13^,^14^ According to the WHO 2018 report, 9 out of 10 people globally are exposed to air containing high levels of pollutants, prompting the organization to label air pollution as “the new tobacco” due to its widespread health impacts and role as a leading cause of premature death and disease.^15^ In 2019, 99% of the world’s population lived in areas that failed to meet the WHO air quality guidelines.^16^ That year alone, pollution was responsible for approximately nine million premature deaths globally, with 6.7 million directly linked to air pollution; 4.2 million deaths were attributed to ambient sources. Strikingly, 89% of ambient air pollution occurred in low- and middle-income countries, highlighting a severe environmental health inequity.^16^,^17^

We aimed to critically examine the mechanisms through which air pollution contributes to cardiopulmonary and systemic diseases, with a particular focus on cardiovascular morbidity, chronic respiratory disorders, and vulnerable populations such as children, pregnant women, and individuals with pre-existing conditions. This review is distinguished from others by integrating recent evidence on air pollution and coronavirus disease 2019 (COVID-19) interactions, highlighting international differences in air quality standards, and discussing the most recent updates to exposure limits. Furthermore, it emphasizes emerging mechanistic insights and evaluates mitigation strategies at both policy and individual levels. By consolidating these perspectives, this review aims to provide a comprehensive, evidence-based framework that shows the significance of air pollution as a global health crisis and identifies critical directions for future research and policy.

## Potential Linking of Air Pollution to Cardiovascular Mortality and Cardiometabolic Risk

The precise mechanisms underlying the cardiovascular system’s heightened sensitivity to air pollution remain incompletely understood but may be partly explained by its direct interface with pulmonary air or its relatively limited detoxification capacity compared to organs like the liver or lungs.^18^ Although the lungs are the primary site of particle deposition, inhaled pollutants can exert harmful effects on distant organs, such as the heart and blood vessels, through several interrelated pathways (**Figures 1** and **2**). Proposed mechanisms include: (1) the release of biological mediators, such as inflammatory cytokines and potentially unidentified signaling molecules, from the lungs into the systemic circulation, (2) activation of sensory receptors in the alveoli that trigger neural reflexes and alter autonomic nervous system activity or stimulate endocrine responses, and (3) the direct translocation of particles or their chemical constituents into the bloodstream, where they may directly impair cardiovascular function. These pathways are not mutually exclusive and are often interconnected, with inflammation and oxidative stress serving as central amplifiers of cardiovascular injury across mechanisms.^19^

**Figure 1:**
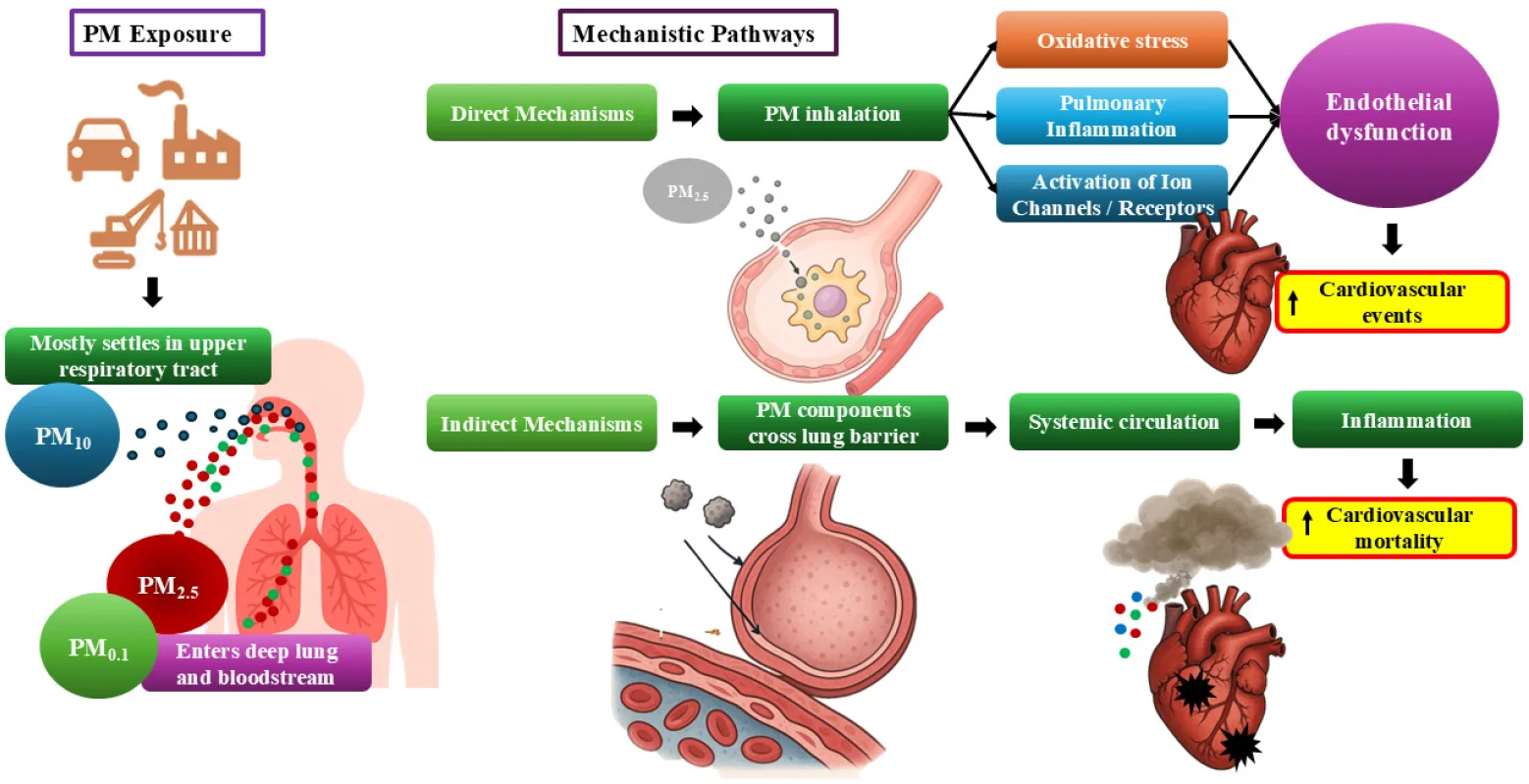
Potential mechanisms of PM exposure on cardiovascular disease mortality. Direct pathways involve PM inhalation, oxidative stress, pulmonary inflammation, and endothelial dysfunction, leading to cardiovascular events. Indirect pathways occur when PM components cross the lung barrier, enter the systemic circulation, and induce systemic inflammation, ultimately contributing to increased cardiovascular mortality. Created with Microsoft PowerPoint and Adobe Photoshop. PM: Particular matter; PM_0.1_: ultrafine PM (mean aerodynamic diameter < 0.1 μm); PM_10_: coarse PM (mean aerodynamic diameter 10–2.5 μm); PM_2.5_: fine PM (mean aerodynamic diameter 2.5–0.1 μm).

**Figure 2:**
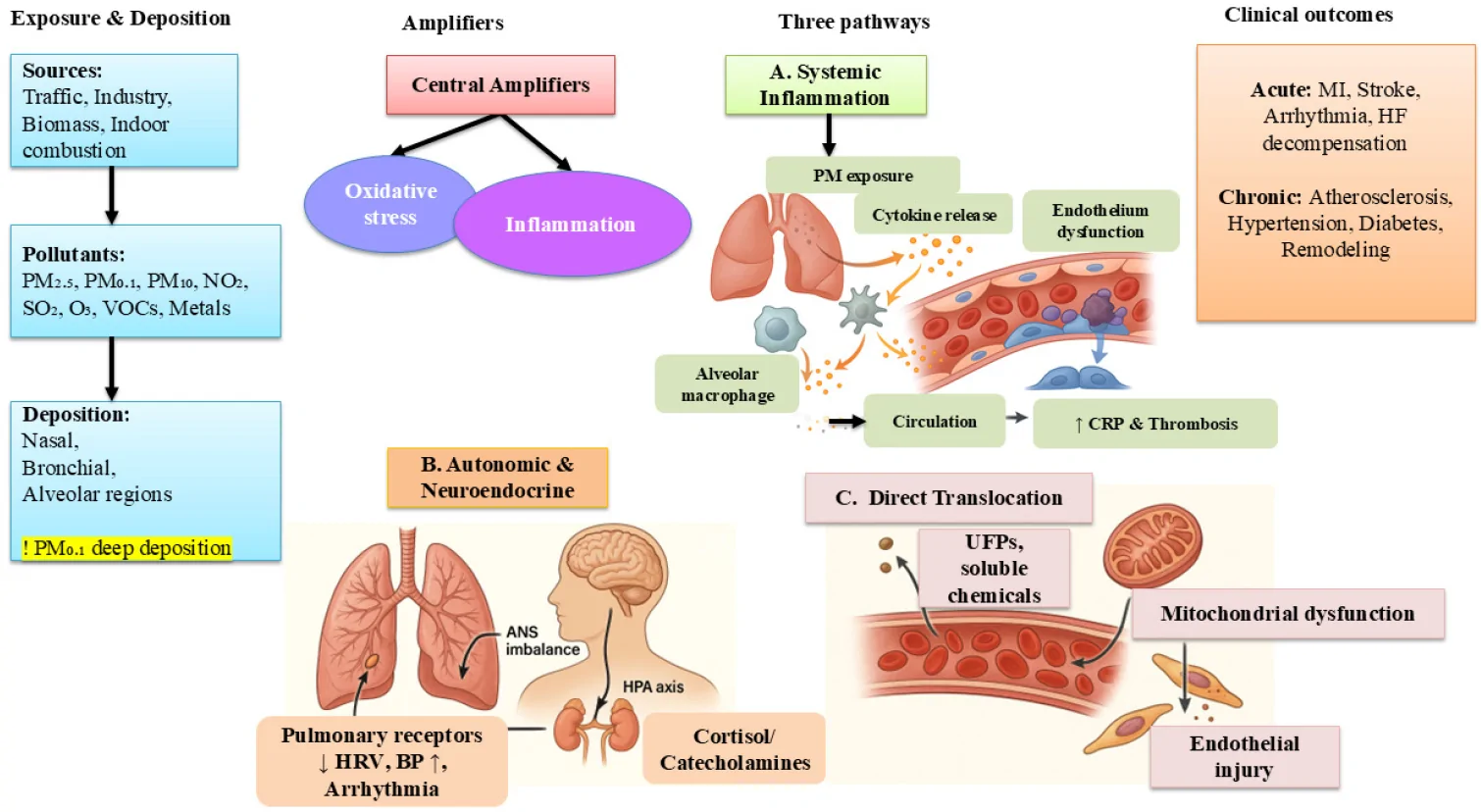
Integrated mechanistic pathways from inhalation to cardiovascular injury. Air pollutants initiate oxidative stress and inflammation that drive systemic inflammation, autonomic imbalance, and direct translocation of particles, ultimately leading to cardiovascular injury and disease. Created with Microsoft PowerPoint and Adobe Photoshop. ANS: Autonomic nervous system; BP: blood pressure; CRP: C-reactive protein; HF: heart failure; HPA: hypothalamic–pituitary–adrenal; HRV: heart-rate variability; MI: myocardial infarction; NO_2_: nitrogen dioxide; O_3_: ozone; PM: particulate matter; PM_0.1_: ultrafine PM (mean aerodynamic diameter < 0.1 μm); PM_10_: coarse PM (mean aerodynamic diameter 10–2.5 μm); PM_2.5_: fine PM (mean aerodynamic diameter 2.5–0.1 μm); SO_2_: sulfur dioxide; UFP: ultrafine particle; VOC: volatile organic compound.

Previous research has largely concentrated on elucidating the cellular mechanisms and biological pathways through which PM contributes to cardiovascular mortality and cardiometabolic risk. Considering the association between PM exposure and adverse cardiovascular outcomes, both direct and indirect mechanisms have been proposed to explain this connection. In general, inhaled PM can directly induce oxidative stress or local inflammation, or activate ion channels and receptors, ultimately leading to endothelial dysfunction and subsequent cardiovascular events. Indirectly, the chemical constituents of PM may enter the circulatory system through interactions with lung cells, triggering systemic inflammation and contributing to cardiovascular mortality.^5^ However, there is biological plausibility that reductions in PM exposure are associated with decreased progression of carotid intima-media thickness, a recognized surrogate marker for cardiovascular risk.^14^,^20^ The differential toxicity of PM fractions also plays a role, as PM_2.5_ and PM_0.1_ particles, due to their smaller diameter, can penetrate deeper into the respiratory tract and reach the bloodstream, whereas PM_10_ particles are more likely to deposit in the upper airways and nasal cavities.^21^ Last but not least, in line with previous research evidence, PM_2.5_ appears to be linked to a higher risk of cardiovascular mortality compared to PM_10_.^22^,^23^,^24^ To be more cellular precise, upon inhalation, PM_2.5_ particles are primarily taken up by alveolar macrophages, leading to impaired pulmonary redox metabolism and the initiation of local inflammation. These events trigger the release of oxidative and inflammatory mediators into the systemic circulation, promoting widespread inflammation further amplified by autonomic nervous system dysfunction. As a result, endothelial dysfunction and vascular inflammation emerge as key features of the systemic biological response to PM_2.5_ exposure. In cardiac tissue, PM_2.5_ impairs mitochondrial respiration, reduces contractile function, and exacerbates myocardial infarction and inflammatory remodeling. Similarly, in both white and brown adipose tissue, PM_2.5_ disrupts redox balance and inflammatory signaling, leading to altered metabolic function. Collectively, these mechanisms contribute to the development of cardiometabolic risk factors such as obesity and type 2 diabetes, ultimately increasing the burden of cardiovascular disease (CVD) morbidity and mortality.^25^

## Hidden Heart Risks of Air Pollution Exposure

### Impact of PM_2.5_ as a standalone exposure

The 2022 American Heart Association Statistical Update indicates that CVD has significantly impacted global health and created a substantial disease burden.^26^ The 2021 Paul Wood Lecture of the British Cardiovascular Society highlighted that air pollution is a significant global health threat, responsible for up to 8.8 million excess deaths annually.^27^ While the Lancet Commission on pollution and health reported that pollution is responsible for approximately 9 million deaths annually, accounting for nearly one in six deaths worldwide.^28^ Importantly, CVD appears to be the leading contributor to this mortality burden.^27^ Given the growing body of evidence linking air pollution exposure to CVD, it is unsurprising that an increasing number of studies and umbrella reviews are being conducted to further investigate this complex and significant relationship.^7^,^29^

Air pollution ranks as the fourth largest modifiable risk factor for CVD globally, according to the Global Burden of Disease Study 2019.^30^ Supporting this, the Lancet Commission on Pollution and Health reported that air pollution was responsible for approximately 21% of all CVD-related deaths in 2015, highlighting its substantial contribution to global cardiovascular mortality.^31^

Over the last quarter-century, extensive evidence has shown that exposure to elevated levels of PM_2.5_ contributes to long-term cardiovascular damage. This cumulative physiological burden significantly increases the risk of developing CVD and is a major factor in premature mortality. Although PM_2.5_ has been linked to various adverse health outcomes, 70–90% of deaths related to PM_2.5_ are attributed to cardiovascular causes, reflecting the same mortality pattern associated with tobacco smoke exposure.^32^ Emerging data also indicate that air pollution may play a contributory role in the initial stages of CVD progression.^33^ Over the past 25 years, strong evidence has shown that inhaling high levels of PM_2.5_ induces oxidative stress, triggers inflammation, and activates the autonomic nervous system, effects that accumulate over time and significantly increase the risk of CVD and premature death.^5^,^32^,^34^ While PM_2.5_ exposure is linked to various health conditions, the majority of related deaths (70–90%) are from CVD, similar to patterns seen with smoking.^5^,^34^,^35^

Short-term exposure to PM_2.5_ levels over hours to days has been shown to trigger cardiovascular events, hospital admissions, and even mortality.^36^,^37^ The most pronounced associations between PM_2.5_ exposure and heart failure (HF) have been observed on the same day of exposure, highlighting its role in the acute exacerbation of HF symptoms. This risk appears to be even more pronounced in heart transplant recipients, likely due to their heightened physiological vulnerability.^38^ In contrast, long-term exposure over months to years is associated not only with a higher risk of major adverse cardiovascular events^39^,^40^,^41^,^42^ but also with the accelerated progression of underlying conditions, such as atherosclerosis.^41^ Interestingly, regarding the long-term effects of PM_2.5_ exposure on cardiovascular outcomes, Hayers et al.^43^ found that each 10 µg/m^3^ increase in PM_2.5_ was associated with a 16% higher risk of ischemic heart disease death and a 14% higher risk of stroke death. Moreover, increased cardiovascular mortality was evident even below the current US PM_2.5_ standard of 12 µg/m^3^, indicating adverse effects at lower exposure levels. A meta-analysis by Alexeeff et al.^44^ has observed that a 10 µg/m^3^ increase in PM_2.5_ has been associated with a 23% higher risk of ischemic heart disease mortality, 24% higher cerebrovascular mortality, 13% increased risk of incident stroke, and an 8% increase in incident myocardial infarction. However, the latter was not statistically conclusive. More recently, Wang et al.^45^ noticed that among US Medicare participants, long-term exposure to low levels of PM_2.5_ (< 9 μg/m^3^), NO_2_ (< 75.2 µg/m^3^), and warm-season O_3_ (< 88.2 μg/m^3^) was positively associated with higher rates of stroke-related hospitalizations. These associations were observed in three-pollutant models that accounted for correlations among co-occurring air pollutants and controlled for unmeasured confounding using negative control exposures.

Considering arteriosclerosis, Kaufman et al.^41^ found that each 5 μg/m^3^ increase in PM_2.5_ concentration was linked to an accelerated progression of coronary artery calcium by 4.1 Agatston units per year (95% confidence interval: 1.4–6.8), reinforcing its role in advancing subclinical atherosclerosis. Similarly, Cesaroni et al.^42^ reported that a 5 μg/m^3^ increase in estimated annual mean PM_2.5_ was associated with a 13% higher risk of coronary events. Exposure to PM_10_ or concentrated PM_2.5_ may lead to increased vascular and macrophage inflammation, enhanced plaque development, elevated tissue factor levels, smooth muscle cell accumulation, and altered vasomotor tone, all of which are features indicative of more vulnerable plaques.^46^ These effects are likely driven by systemic inflammation, which boosts inflammatory cell recruitment while impairing the mobilization of angiogenic cells from the bone marrow.^46^,^47^

In addition, higher exposure to PM_2.5_ has been consistently linked to vascular calcification. Notably, this association persists even at average exposure levels around 15 μg/m^3^ or lower, underscoring the cardiovascular risks posed by relatively low concentrations of PM_2.5_.^48^ Moreover, increased levels of ambient PM_2.5_ have been associated with a rise in hospital admissions and mortality related to HF. PM_2.5_ may directly impair heart function^49^ and significantly increase the risk of coronary heart disease, HF, and stroke.^50^ In a general middle-aged population, long-term exposure to residential PM_2.5_ was associated with reduced left ventricular function, suggesting structural remodeling that autonomic, vascular, or electrophysiological factors may influence.^51^ Moreover, higher exposure to PM_2.5_ has been associated with increased odds of developing circadian syndrome, with a clear exposure–response relationship observed. Circadian rhythms partially explain the relationship between PM_2.5_ and CVD, suggesting that disruptions to these rhythms may be a key biological mechanism through which air pollution contributes to cardiovascular risk.^52^

### Distinct effects of PM_2.5_ *versus* other air pollutants

Studies have linked long-term exposure to PMs with adverse cardiovascular effects, such as ischemic heart disease, stroke risk and mortality.^43^ In a previous meta-analysis,^53^ it was found that long-term exposure to PMs is associated with increased risk of stroke incidence and mortality. A 10 μg/m^3^ increase in PM_1.0_ was linked to a 6.1% higher risk of stroke events and 8.0% higher stroke mortality, while a 5 μg/m^3^ increase in PM_2.5_ was associated with 6.4% and 12.5% increases, respectively. Stronger associations were observed in North America and Europe, with more variability in Asian studies. Likewise, a recent systematic review and meta-analysis synthesizing data from 100 studies across 20 countries provided robust evidence of adverse associations between air pollution and HF.^54^ Both short- and long-term exposures to key pollutants, including PM_2.5_, PM_10_, NO_2_, SO_2_, and CO, were significantly linked to increased risks of HF hospitalization, incidence, and mortality. Notably, short-term exposure to PM_2.5_ and PM_10_ was associated with a 1.8% and 1.6% increase in HF risk per 10 μg/m^3^, respectively, with stronger associations observed over lag periods of up to 2 days. Long-term exposure posed even greater risk, particularly for PM_2.5_, with a relative risk of 1.748 per 10 μg/m^3^ increase. These effects were more pronounced in low- and middle-income countries, highlighting the heightened vulnerability in these populations and underscoring the global urgency for targeted air quality interventions.

HF has also been associated with air pollutants such as PM_10_, NO_2_ and black carbon. In a cross-sectional study of 671 randomly selected Flemish adults, long-term residential exposure to low levels of air pollutants, including black carbon, PM_2.5_, PM_10_, and NO_2_, was significantly associated with subclinical impairments in left ventricular function.^51^ Higher pollutant levels correlated with reduced systolic strain and strain rate and lower diastolic mitral E and a’ peak velocities, suggesting early cardiac dysfunction. Geographic patterns aligned with pollution gradients, and path analysis indicated systemic inflammation may mediate the effects of black carbon. In alignment with these results, Vadugaranathan et al.^55^ by analyzing over 6000 cases of acute cardiovascular hospitalizations in Brescia, Italy, from 2004 to 2007, found that short-term increases in PM_10_ levels (even below the European Union safety threshold of 50 μg/m^3^) were significantly associated with higher rates of hospital admissions for various acute cardiovascular events. For every 1 μg/m^3^ increase in PM_10_ (measured on the same day), there was a modest but consistent rise in the risk of composite cardiovascular events, acute HF, atrial fibrillation, and other related conditions. Similar patterns were observed when using 3-day moving averages. Notably, the increased risk did not vary significantly above or below the European Union limit or across different age and gender groups, indicating that even low-level PM_10_ exposure poses a public health risk. Moreover, a recent meta-analysis by Strobe et al.^56^ showed that each 10 μg/m^3^ increase in PM_10_ concentration is linked to a higher risk of myocardial infarction (odds ratio = 1.01; 95% confidence interval: 1.00–1.02) both on the day of exposure and the day after exposure. While long-term exposure to PM, particularly PM_2.5_ and PM_10_, significantly increases the risk of transitioning from asthma to CVD, suggesting a notable impact on the CVD trajectory.^57^

Furthermore, controlled human exposure studies offer direct mechanistic insights into the cardiovascular effects of air pollution, particularly by elucidating the roles of NO and NO_2_ in cardiovascular health. For example, Langrish et al.^58^ demonstrated that short-term diesel exhaust inhalation disrupts vascular homeostasis; although plasma nitrite levels increased, reflecting enhanced NO generation, this failed to compensate for excess NO consumption. While endothelium-dependent and -independent vasodilation were preserved, diesel exhaust exposure augmented systemic blood pressure and arterial stiffness during NO synthase inhibition, suggesting that reduced NO bioavailability contributes to air pollution–induced vascular dysfunction. Complementing these findings, a clinical trial (NCT00845169) was designed to investigate the contribution of specific nitric oxide synthase isoforms to diesel exhaust–related vascular changes, randomizing healthy volunteers (18–40 years) to 1-hour exposures of diesel exhaust (300 µg/m^3^) or filtered air during intermittent exercise, with vascular responses assessed by forearm venous occlusion plethysmography during intrabrachial infusion of nitric oxide synthase inhibitors and norepinephrine. Similarly, another clinical trial (NCT00774514) sought to examine the vascular impact of acute NO_2_ exposure, randomizing healthy male volunteers to 1-hour exposure of filtered air or 4 parts per million (ppm) NO_2_ during intermittent exercise, with plethysmographic assessment during infusion of acetylcholine, sodium nitroprusside, bradykinin, and verapamil. At present, no results from either of these clinical trials have been published or posted on ClinicalTrials.gov.

Additionally, the U.S. Environmental Protection Agency (EPA) has concluded that the evidence for a causal link between long-term O_3_ exposure and cardiovascular effects remains only suggestive.^59^ However, experimental studies have demonstrated that O_3_ exposure can alter biomarkers associated with fibrinolysis, oxidative stress, and inflammation, mechanisms closely tied to the development of CVD.^60^,^61^,^62^,^63^ Still, epidemiologic findings remain inconsistent and current evidence is insufficient to establish a definitive causal link between O_3_ exposure and CVD. Epidemiological studies across the US,^64^,^65^ Europe,^66^,^67^ Canada,^68^,^69^ Latin America,^70^,^71^ and China^72^,^73^ have linked short-term O_3_ exposure to increased CVD mortality, with meta-analyses in China estimating 0.62–1.28% higher risk per 10 µg/m^3^ increase.^74^,^75^,^76^,^77^ Acute exposure has also been associated with a 0.72% rise in total CVD morbidity.^78^ Evidence for long-term effects is less consistent: while some cohorts observed 1.3–7.5% higher CVD mortality per 10 µg/m^3^ increase in warm-season O_3_,^79^,^80^,^81^,^82^,^83^,^84^,^85^,^86^ others reported null results,^87^,^88^,^89^,^90^,^91^,^92^,^93^ with pooled risks ranging from 0.51% to 1.87%.^94^,^95^ More recent Chinese cohorts reported stronger effects, with a 9.3–11% increase in CVD mortality per 10 µg/m^3^,^96^,^97^ underscoring both the potential importance of O_3_ and the need for further robust longitudinal studies.

Significant uncertainties still remain, including challenges in exposure assessment, differences in exposure intensity, population characteristics, and potential interactions with co-pollutants. The biological pathways through which O_3_ affects the cardiovascular system are not yet fully understood, with gaps in knowledge about the timing and interplay of underlying mechanisms. As a result, the existing research evidence does not yet provide a comprehensive explanation of how O_3_ exposure contributes to cardiovascular injury, limiting the interpretation of epidemiological findings.^98^

Moreover, there is currently no established standard for long-term exposure to ambient O_3_. Consequently, further investigation is warranted. In a recent large prospective cohort study by Liang et al.^99^ which utilized data from two large cohort studies, a notable association was found between long-term exposure to maximum daily 8-hour average O_3_ and an increased incidence of CVD, particularly heart disease and hypertension. In the China Health and Retirement Longitudinal Survey cohort, each 10 parts per billion (ppb) increase in long-term exposure to maximum daily 8-hour average O_3_ was linked to a 31% higher risk of heart disease, with a population-attributable fraction of 13.79%. In the China Family Panel Studies cohort, the same increase in O_3_ exposure was associated with a 7% rise in overall CVD risk and a 10% increase in hypertension risk, with population-attributable fractions of 3.53% and 5.11%, respectively. Stronger associations were observed among males, urban residents, and individuals living in Southern China, underscoring the need for continued efforts to control O_3_ pollution.

## Mechanisms and Impact of Air Pollution on Cardiopulmonary and Respiratory Health

Extended exposure of individuals and groups to air pollutants (PM_2.5_, PM_10_, NO_2_, NO_x_, and SO_2_) may also be a significant risk factor for cardiopulmonary diseases and mortality in high-risk populations with preserved ratio impaired spirometry.^100^ One of the earliest landmark studies in this field, conducted across six major U.S. cities, demonstrated a 26% higher risk of mortality from cardiopulmonary diseases among individuals residing in the most polluted cities compared to those in the least polluted areas. The research identified a positive correlation between air pollution and mortality from lung cancer and cardiopulmonary diseases, while no significant link was found regarding mortality from other causes. The strongest associations were related to exposure to PM_2.5_, especially those particles containing sulfates.^101^ These findings have been corroborated by subsequent research, including a large prospective cohort study of 500,000 adults from all 50 U.S. states, which reported a 9% increase in cardiopulmonary mortality and an 18% increase in lung cancer mortality for every 10 µg/m^3^ rise in PM_2.5_ concentration.^102^

Recent large-scale cohort studies in Europe and the UK have broadened the evidence linking long-term exposure to air pollutants (PM_2.5_, NO_2_, and black carbon) with a wide spectrum of health outcomes beyond cardiorespiratory diseases, including diabetes, dementia, and psychiatric disorders.^103^,^104^ In the Danish nationwide cohort, a 5 µg/m^3^ increase in PM_2.5_ was associated with an ~11% rise in all-cause mortality and higher risks across multiple disease-specific outcomes.^103^ Similarly, UK Biobank data demonstrated that PM_2.5_ consistently increased the risk of progression from health to chronic lung disease, multimorbidity, and mortality over long-term follow-up.^104^ Overall, these studies show the systemic impacts of air pollution on multiple organ systems and reinforce PM as a major driver of morbidity and mortality, including adverse respiratory outcomes such as asthma and chronic obstructive pulmonary disease (COPD).^105^

Although there is strong epidemiological evidence linking PM exposure to the development and progression of COPD, the mechanistic understanding of the cellular and molecular pathways involved in PM-induced COPD remains limited. To address this gap, researchers have developed both in vitro and in vivo models to investigate the underlying pathophysiological mechanisms of PM-induced lung disease.^106^,^107^,^108^,^109^ A summary of this finding is shown in **Figure 3**. A large-scale epidemiological study conducted in the United Kingdom, comprising 303,887 participants, demonstrated that a 5 μg/m^3^ increase in ambient PM_2.5_ concentration was significantly associated with a reduction of 83 mL in forced expiratory volume in 1 second and 62 mL in forced vital capacity, alongside a 52% increase in the prevalence of COPD.^110^ While a cohort study from Norway involving 57,000 participants reported that exposure to 5.8 µg/m^3^ increase in NO_2_ levels has been linked to an 8% rise in COPD incidence.^111^

**Figure 3:**
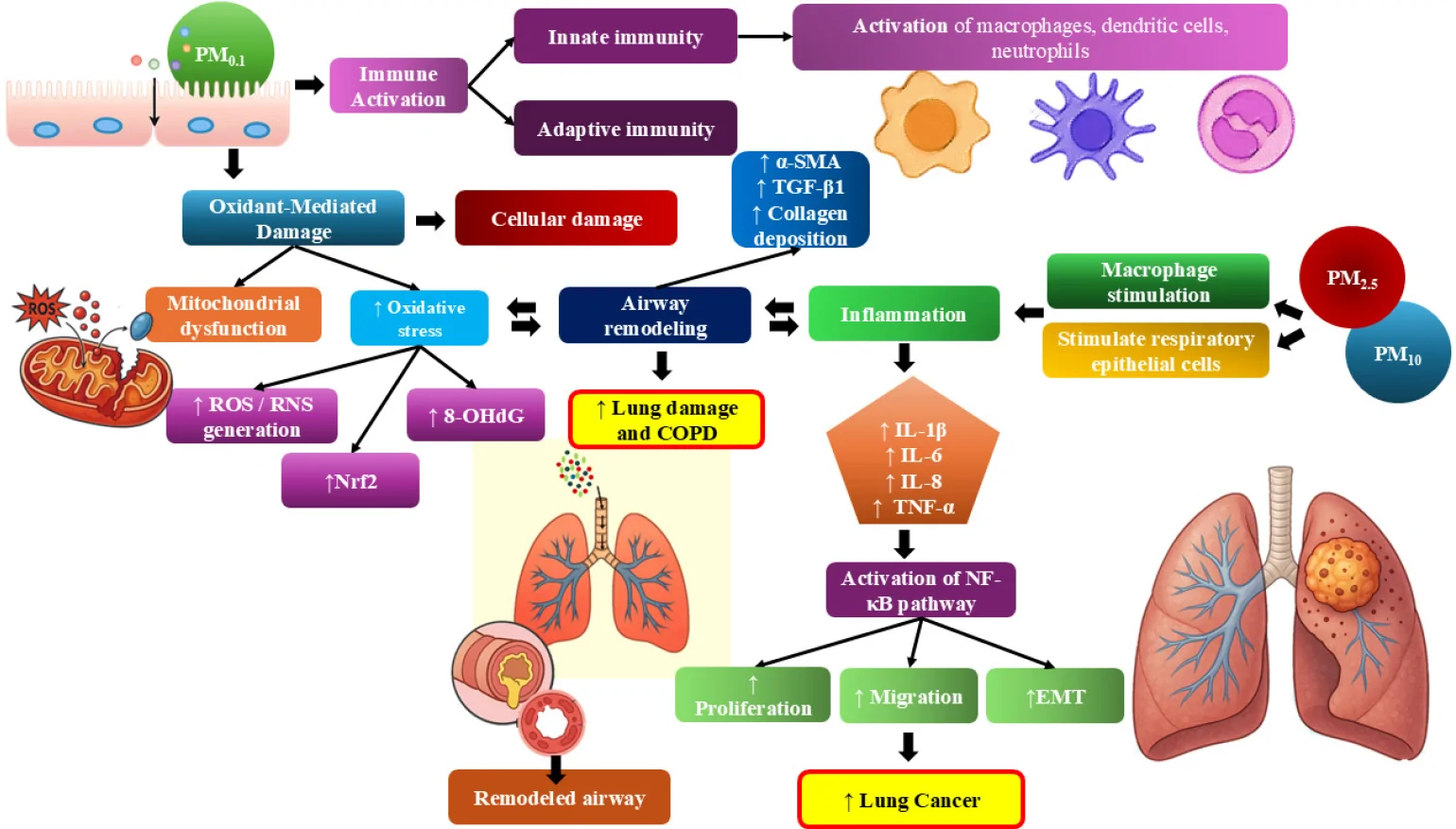
The linking of air pollution–induced particulate matter and lung dysfunction, COPD, and lung cancer. PM triggers immune activation and oxidant-mediated damage, leading to mitochondrial dysfunction, oxidative stress, and airway remodeling. These processes promote inflammation, activation of the NF-κB pathway, and stimulation of macrophages and epithelial cells. The combined effects result in lung tissue damage, chronic airway remodeling, COPD progression, and, ultimately, increased risk of lung cancer. Created with Microsoft PowerPoint and Adobe Photoshop. ↑: Increase; 8-OHdG: 8-hydroxy-2′-deoxyguanosine; COPD: chronic obstructive pulmonary disease; EMT: epithelial-mesenchymal transition; IL: interleukin; NF-κB: nuclear factor kappa B; Nrf2: nuclear factor erythroid 2-related factor 2; PM: particular matter; PM_0.1_: ultrafine PM (mean aerodynamic diameter < 0.1 μm); PM_10_: coarse PM (mean aerodynamic diameter 10–2.5 μm); PM_2.5_: fine PM (mean aerodynamic diameter 2.5–0.1 μm); RNS: reactive nitrogen species; ROS: reactive oxygen species; TGF-β1: transforming growth factor beta 1; TNF: tissue necrosis factor; α-SMA: alpha smooth muscle-actin.

Furthermore, PM_0.1_ is readily deposited in the airways and centriacinar regions of the lung, contributing to the development and exacerbation of airway and respiratory diseases, including asthma and COPD. PM_0.1_-induced health effects are largely mediated by oxidant-induced cellular damage, including the generation of reactive oxygen species, oxidative stress, and the activation of both innate and adaptive immune responses.^112^,^113^ PM_0.1_ has been associated with respiratory symptoms such as cough and reduced peak expiratory flow,^114^ as well as increased use of medications^115^ and higher rates of hospital admissions among individuals with asthma.^116^ Elevated PM_0.1_ levels have also been linked to an increase in clinical visits for respiratory illness.^117^ While many studies report a positive association between PM_0.1_ exposure and asthma symptoms, a large cohort study involving over 1 million adult residents in Toronto found no significant association between long-term PM_0.1_ exposure and respiratory disease after adjusting for PM_2.5_, NO_2_, and other covariates.^118^ Similarly, an Australian controlled study reported no independent association between particle number concentration and respiratory symptoms, asthma diagnosis, or lung function. However, PM_0.1_ exposure was associated with elevated inflammatory markers in atopic individuals.^119^ These mixed findings highlight that global evidence on PM_0.1_ remains limited, and important knowledge gaps persist. For example, it is unclear why PM_0.1_ is not more strongly associated with lung cancer incidence, given that diesel exhaust is a known carcinogen and diesel vehicles are a major source of PM_0.1_.^120^,^121^,^122^ One possible explanation is that rapid condensation and agglomeration of PM_0.1_ into larger particles concentrate carcinogenic compounds into fractions such as PM_2.5_, which has consistently been associated with lung cancer.^123^ In addition, long-term residential estimates may not adequately capture personal PM_0.1_ exposures, as other microenvironments such as transportation contribute substantially.^122^ Future research should therefore incorporate mobility information (e.g., home and workplace locations) into exposure assessment to better address the high spatial variability of PM_0.1_. Furthermore, comparability across studies is hampered by differences in exposure modeling, which may introduce bias depending on how well residential estimates reflect true long-term exposures.^81^

Moreover, according to a recent review by Berg et al.^8^ together with findings from the Global Burden of Diseases, Injuries, and Risk Factors Study (GBD) 2017 comparative risk assessment, air pollution has emerged as the second leading cause of lung cancer globally,^124^ with a nearly 30% increase in attributable lung cancer deaths since 2007,^125^ coinciding with rising pollution levels and declining smoking rates. The International Agency for Research on Cancer classified outdoor air pollution and PM_2.5_ as carcinogenic to humans in 2013.^126^,^127^ Exposure to PM_2.5_ plays a significant role in the pathogenesis of lung cancer, potentially through multiple biological mechanisms, including chronic inflammation, oxidative stress, DNA damage, and epigenetic modifications.^128^ PM_2.5_ compounds stimulate respiratory epithelial cells and macrophages to release pro-inflammatory mediators, including interleukin (IL) 1β, IL-6, IL-8, and tissue necrosis factor α. This cytokine release activates the nuclear factor kappa B signaling pathway, which in turn promotes increased cellular proliferation, migration, and epithelial–mesenchymal transition, thereby contributing to an elevated risk of tumor development and progression^129^,^130^,^131^,^132^,^133^,^134^ (**Figure 3**). Air pollution not only increases the risk of lung cancer but also worsens outcomes and acts synergistically with smoking. Current lung cancer risk models, however, do not account for air pollution due to challenges in accurately estimating long-term exposure.^8^ In a recently published study by Hill et al.,^135^ it was noted that PM_2.5_ promotes lung cancer by acting on pre-existing oncogenic mutations in normal lung tissue. Analysis of 32,957 epidermal growth factor receptor (*EGFR*)-driven lung cancer cases revealed a strong association between PM_2.5_ exposure and cancer incidence, particularly in never-smokers. Mouse models showed that PM_2.5_ triggers macrophage infiltration and IL-1β release, inducing a tumor-promoting state in *EGFR*-mutant alveolar cells. Mutational profiling further identified *EGFR* and Kirsten rat sarcoma viral oncogene homolog (*KRAS*) driver mutations in normal lung tissue, supporting that PM_2.5_ is a tumor promoter and emphasizing the need for air pollution control to reduce cancer risk.

## Unequal Exposure, Unequal Outcomes: Air Pollution and Cardiorespiratory Health in Vulnerable Groups

### Studies in pregnancy

#### Human studies

In a recent review, Decrue et al.^136^ showed the significant impact of ambient air pollution on maternal cardiovascular health during pregnancy, identifying pregnant women as a particularly vulnerable group due to complex physiological changes and heightened susceptibility of the fetoplacental system. In this context, several studies in humans have investigated the impact of air pollution exposure on placental vascularization and function, yielding conflicting results regarding placental vascular resistance to blood flow in the uterine circulation.^137^,^138^,^139^,^140^ The results of recent studies in humans are shown in **Table 1**.^137^,^138^,^139^,^140^,^141^,^142^

**Table 1:** Clinical research on the effects of air pollution on placental circulation

Study	Year	Location	Study design	Sample size & methodology	Main results
Van de Hooven et al.^137^	2012	Rotterdam, Netherlands	Prospective cohort study	7801 pregnant women with a mean gestational stage 14.4 (10.2–29.5) wk. PM_10_ and NO_2_ levels were estimated at maternal residences using dispersion modeling. Angiogenic markers (PlGF, and sFlt-1), uterine/umbilical artery Doppler indices, third-trimester notching, placenta weight, and birth weight were assessed across pregnancy.	Higher PM_10_ and NO_2_ exposure was linked to lower maternal PlGF and sFlt-1 in the 2^nd^ trimester, and to an anti-angiogenic profile in cord blood. NO_2_ was associated with 3^rd^ trimester notching and both pollutants were linked to reduced placental weight, but not to consistent changes in resistance indices or placenta-to-birth weight ratio.
Carvalho et al.^138^	2016	São Paulo, Brazil	Part of a prospective cohort called Procriar	336 pregnant women, with a mean gestational stage of 39.4 ± 1.3 wk were measured during each trimester using passive personal samplers for NO_2_ and O_3_.	O_3_ exposure in São Paulo was associated with trimester-specific changes in umbilical artery PIs.
				Fetal weight and Doppler velocimetry data were also obtained.	Higher PIs in the 2^nd^ trimester (↑ placental resistance) and lower PIs in the 3^rd^ trimester (↓ placental resistance), suggesting a dynamic impact on placental vascular function.
Contreras et al.^139^	2018	Los Angeles, CA, USA	Part of a prospective study called Behavior in Pregnancy Study	Real-time Doppler velocimetry was performed on 556 pregnant women (< 20 wk gestation), alongside measurements of traffic-related air pollution exposure using CALINE4 (NO_x_, NO, and NO_2_).	Exposure to NO_2_ (land use regression) and nitrogen oxides (CALINE4) was associated with increased risk of high uterine artery resistance in late pregnancy.
				Smoking history during pregnancy was also recorded.	Maternal smoking further elevated this risk and was linked to bilateral notching in mid-pregnancy.
Hettfleisch et al.^140^	2017	São Paulo, Brazil	Cross-sectional study	229 low-risk pregnant women were assessed for personal exposure to NO_2_ and O_3_ using passive monitors during the week prior to evaluation.	Increased NO_2_ levels were significantly associated with reduced vascularization index and vascularization flow index in the first trimester, while NO_2_ and O_3_ showed no effect on placental volume or flow index.
Ouidir et al.^141^	2021	Many cities in USA	Multicentral cohort study	2562 pregnant women across 12 clinics were undergone up to five standardized ultrasounds with Doppler.	IQR increases in PM_2.5_, POM, NO_2_, and related pollutants were linked to higher uterine artery resistance and lower risk of systolic notch. O_3_ reduced umbilical artery resistance and abnormal flow risk, while elemental carbon and POM increased abnormal flow risk.
				Exposure to 11 air pollutants was estimated for an hour and 2 h prior to each scan using National Air Quality Forecast Capability reanalysis data.	
Cahuana-Bartra et al.^142^	2022	Barcelona, Spain	Prospective cohort	101 pregnant women. Personal NO_2_ exposure (wk 28–32) was measured using diffusion tubes. Placental function was assessed at wk 32 via Doppler (uterine, umbilical, and cerebral artery PIs, CPR, and ductus venosus PI).	Higher personal NO_2_ exposure was significantly linked to lower mean uterine artery PI and showed a suggestive inverse association with cerebroplacental ratio.

CALINE4: A dispersion model for predicting air pollutant concentrations near roadways; CRP: C-reactive protein; IQR: inter-quartile range; NO: nitric oxide; NO_2_: nitrogen dioxide; NO_x_: nitrogen oxide; O_3_: ozone; PIs: pulsatility indices; PlGF: placental growth factor; PM: particular matter; PM_10_: coarse PM (mean aerodynamic diameter 10–2.5 μm); PM_2.5_: fine PM (mean aerodynamic diameter 2.5–0.1 μm); POM: primary organic matter; sFlt-1: soluble Fms-like tyrosine kinase 1.

Furthermore, prenatal exposure to air pollution has been linked to congenital heart defects (CHD), with findings suggesting that exposure to O_3_ and NO_2_ during cardiac embryogenesis increases CHD risk, especially among overweight women.^143^ Higher maternal exposure to PM_2.5_ during the preconception period has been linked to increased risk of CHD in offspring. According to Yuan’s study,^144^ a 10 μg/m^3^ increase in PM_2.5_ was associated with a 2% higher risk of CHD, with septal defects being most affected. The association was stronger in younger mothers and those in northern or low-income areas. These findings align with a population-based cohort study of over 1.2 million births in North Carolina (2003–2015), which found that prenatal exposure to PM_2.5_ and O_3_ during early pregnancy may increase the risk of CHD, specifically tetralogy of Fallot and atrioventricular septal defect.^145^ However, except for these negative consequences, air pollution increases the risk of pregnancy-induced hypertensive disorders,^146^ while PM exposure in early pregnancy may influence fetal cardiac response and has been significantly associated with increased risk of false-positive fetal heart rate monitoring results, leading to unnecessary interventions based on inaccurate fetal heart rate reading.^147^ Interestingly, Grevendonk et al.^148^ by utilizing data from the ENVIRONAGE (ENVIRonmental influence ON early AGEing) birth cohort investigated the impact of PM_10_ and PM_2.5_ exposure during pregnancy on mitochondrial oxidative DNA damage, as measured by 8-hydroxy-2′-deoxyguanosine levels in maternal and umbilical cord blood. The findings revealed that higher exposure to PM_10_ and PM_2.5_ during pregnancy, particularly in the third trimester, was significantly associated with increased mitochondrial 8-hydroxy-2′-deoxyguanosine levels in maternal blood. Notably, PM_10_ exposure during the first and second trimesters was also positively associated with elevated 8-hydroxy-2′-deoxyguanosine in umbilical cord blood. These results suggest that exposure to ambient particulate air pollution during critical windows of pregnancy contributes to increased systemic oxidative stress at the mitochondrial level in both mothers and their newborns, with further cardiorespiratory negative effects.

While the effects of air pollution on systemic blood pressure are relatively minor, it remains uncertain whether this impact significantly contributes to the development of preeclampsia. However, a large-scale study in the U.S. involving over 5 million pregnancies found a significant association between maternal exposure to PM_2.5_ and hypertensive disorders during pregnancy.^149^ Specifically, the study reported an adjusted odds ratio of 1.10 (95% confidence interval: 1.08–1.12) for every 5 μg/m^3^ increase in average PM_2.5_ exposure throughout pregnancy. This association was stronger among white mothers and varied by census division and county-level poverty, indicating potential demographic and regional disparities. A sublinear dose-response relationship was observed, with a threshold around 9 μg/m^3^. Furthermore, an estimated 8.1% (ranging from 6.8% to 9.4%) of cases of hypertensive disorders were attributable to PM_2.5_ exposure, highlighting the significant public health implications and guiding policy strategies for regulating air quality.

#### Animal studies

Furthermore, studies on animals have shown significant results, although they are less useful in understanding how air pollution affects adverse birth outcomes in humans. For instance, research involving rodents has shown that maternal exposure to diesel exhaust or diesel exhaust particles (≥ 300 μg PM/m^3^) during or before gestation can result in several adverse effects on placentation. These effects include decreased vascularization and perfusion,^150^ injuries linked to increased embryo resorption,^151^ and a reduction in the number of cells responsible for maintaining structural integrity.^152^ A previous study by Gorr et al.^153^ demonstrated that pregnant mice exposed to PM_2.5_ developed cardiac dysfunction in their offspring after birth. This included a reduced ejection fraction and an increased ventricular diameter. These findings were further supported by studies showing ventricular dysfunction and prolonged action potentials in isolated cardiomyocytes. To be more precise, Tanwar et al.^154^ found that in utero PM_2.5_ exposure induces inflammation, matrix remodeling, and Ca^2+^ protein changes, leading to adult cardiac dysfunction. Additionally, short-term exposure to ultrafine particles resulted in reduced contractility and isovolumic pressure.^155^ Meanwhile, exposure of rats to PM_2.5_ (600 μg/m^3^ for 60 minutes) before and/or during pregnancy negatively impacts placental development and function. It reduces placental levels of angiogenic factors, indicating that air pollution may hinder trophoblast invasion and the development of placental blood vessels. This impairment could potentially restrict fetal nutrition and growth.^156^

Moreover, O_3_ exposure during early pregnancy impairs fetal growth in rats, likely through vascular and metabolic disruptions. To be more precise, even brief exposure to 0.8 ppm O_3_ can increase uterine arterial resistance and alter maternal metabolism, leading to reduced fetal weight, lean mass, and fat mass.^157^ Additionally, O_3_ can specifically reduce male fetal weight and decrease uterine resistance by mid-gestation, while noise exposure increases resistance without affecting growth.^158^ Bongaerts et al.^159^ found that carbon particles from maternally inhaled diesel exhaust can cross the placenta and accumulate in fetal organs, including the heart, liver, lungs, kidneys, and gonads. Exposed fetuses had significantly higher carbon particle loads than controls, and multivariate analysis clearly distinguished the two groups based on carbon particle levels and fetoplacental biometry. While no clear sex-specific effects were observed, a possible interaction between exposure and fetal sex may exist. These findings suggest that prenatal air pollution exposure may disrupt fetal development and contribute to long-term health risks.

#### In vitro studies

Numerous *in vitro* studies have sought to elucidate the mechanisms by which air pollution alters placental structure and function, thereby providing insight into cellular and molecular responses under controlled experimental conditions. Familari et al.^160^ reported that first-trimester trophoblast cells rapidly internalize urban PM, resulting in decreased human chorionic gonadotropin beta subunit and increased IL-6 secretion, indicative of impaired endocrine function and inflammation. Low-dose PM exposure was shown to reduce cell growth and alter protein expression linked to oxidative stress, endoplasmic reticulum stress, and disrupted cellular homeostasis, changes that mirror pathways implicated in pre-eclampsia and suggest a role for ambient PM in placental dysfunction and potential biomarker development. Similarly, Nääv et al.^161^ demonstrated that first-trimester trophoblast cells internalize PM_2.5_ within 24 hours, with particles localizing to mitochondria and inducing structural damage, including cytoskeletal disruption, organelle swelling, and loss of membrane integrity. Exposure also led to decreased human chorionic gonadotropin and increased progesterone and IL-6 secretion, along with elevated cytotoxicity, protease activity, and oxidative stress markers; however, mitochondrial respiration and mass remained unaffected. While Deval et al.^162^ further observed that benzo(a)pyrene (BaP) and cerium dioxide nanoparticles, common air pollutants detected in maternal blood, modulate key signaling pathways in human placental cells. At non-toxic concentrations, BaP activated the aryl hydrocarbon receptor pathway, inducing xenobiotic metabolism, phosphorylated form of the histone-H2AX (γ-H2AX) DNA damage, and stress responses via p53 and p21. Co-exposure with cerium dioxide nanoparticles reproduced most BaP-induced effects but attenuated γ-H2AX induction, suggesting a modulation of BaP’s genotoxicity. Additionally, cerium dioxide nanoparticles alone or in combination with BaP reduced peroxiredoxin overoxidation levels, indicating a potential antioxidant role. These findings provide new insights into placental cellular responses to individual and combined pollutant exposures.

### Studies in newborns, infants and children

Approximately 93% of the world’s children under the age of 15 (around 1.8 billion) are exposed to polluted air, which puts them at significant risk of serious health issues during essential stages of their development. According to the WHO, both ambient and household air pollution are linked to one in 10 deaths in this age group, resulting in approximately 600,000 fatalities.^163^ Fetuses, infants, and children are especially vulnerable to air pollution, which significantly affects their cardiorespiratory health due to various physiological, behavioral, and environmental factors.^164^ Scientific evidence from epidemiologic and experimental studies indicates that high exposure to air pollutants (i.e., PM_2.5_ and PM_0.1_) is linked to adverse birth outcomes and long-term effects on children’s respiratory function, immune response, brain development, and cardiometabolic health.^165^ Interestingly, results from a recent nationwide longitudinal study by Kuo et al.^166^ showed that prenatal exposure to several pollutants, specifically CO, NO, NO_2_, and NO, was positively associated with increased Kawasaki disease (KD) incidence, with a consistent dose-dependent relationship observed for both prenatal and postnatal exposure. O_3_, on the other hand, showed a negative or no significant association. These results are aligned with those from previous research, indicating that exposure to air pollutants may trigger KD, despite the fact that the etiology of KD still remains unknown. For instance, Kwon et al.^167^ in a large-scale national study conducted in South Korea, found a positive association between daily fluctuations in ambient air pollution and the incidence of KD in children under 5 years of age. More specifically, exposure to PM_2.5_ and SO_2_ prior to disease onset was significantly associated with an increased risk of KD.

Additionally, traffic-related pollutants may raise allergic susceptibility in young children and potentially increasing the risk of KD.^168^ Exposure to traffic-related air pollutants has been associated with an increased susceptibility to allergies in children, particularly conditions such as asthma, allergic rhinitis, and atopic dermatitis. Notably, children with allergic diseases have a higher risk of developing KD. Tsai et al.^168^ and Wei et al.^169^ reported that allergic conditions in early childhood are associated with a higher incidence of KD. Similarly, Huang et al.^170^ highlighted overlapping immune dysregulation in both KD and allergic diseases, suggesting shared environmental and immunological pathways. However, heavy traffic exposure not only influences the KD risk but also harms children’s respiratory health. A cross-sectional study of over 4000 schoolchildren in Munich found that higher road traffic density was linked to reduced lung function and more respiratory symptoms. For every 25,000-car increase in daily traffic, peak and maximum expiratory flow dropped by 0.71% and 0.68%, respectively. Recurrent wheezing and dyspnea also increased, while no significant associations were found for asthma or bronchitis.^171^ In low- and middle-income countries, 98% of children under the age of 5 years are exposed to PM_2.5_ at levels that exceed the WHO air quality guidelines. Similarly, approximately 52% of children under 5 years old in high-income countries are also exposed to PM_2.5_ levels that surpass these guidelines.^165^ The frequent exposure to air pollutants has been correlated with acute upper and lower respiratory infections, as well as pneumonia and asthma.^172^ Additionally, prenatal exposure to air pollution can negatively impact fetal growth and lung development through mechanisms like increased inflammation, oxidative stress, and placental dysfunction. There is also emerging evidence that air pollution affects genetic and epigenetic processes vital for lung and immune system development.^173^,^174^,^175^,^176^

In a recent cross-sectional and retrospective cohort study, Lu et al.^177^ observed that intrauterine and post-natal exposure to PM_2.5_, PM_10_, and PM_1.0_ were linked to increased risk of doctor-diagnosed pneumonia in children. The second and third trimesters and the first year of life were identified as critical exposure windows. PM_2.5_ during pregnancy and PM_10_ after birth showed the strongest associations, with early-life traffic-related pollution further increasing risk. Similarly, Zhou et al.^178^ found significant associations between exposure to major air pollutants, such as PM_2.5_, PM_10_, NO_2_, and SO_2_, and increased hospitalizations for asthma and pneumonia among children under 5 years old in Ningbo, China. While Ünal et al.^179^ observed that pediatric respiratory admissions increased with elevated levels of PM and gaseous pollutants, highlighting a clear temporal relationship between pollution exposure and health outcome. More recently, Johnson et al.^180^ investigated the impact of prenatal exposure to air pollution, specifically PM_2.5_ and NO_2_, on neonatal respiratory distress using data from the Maternal-Infant Research on Environmental Chemicals (MIREC) study. They found no associations between air pollution and physician-diagnosed respiratory distress, oxygen therapy, or neonatal intensive care unit admissions. However, prenatal exposure to PM_2.5_ was significantly linked to more severe outcomes, including a higher likelihood of assisted ventilation, multiple clinical interventions, and increased use of systemic antibiotics. Additionally, exposure to NO_2_ was associated with a modest increase in antibiotic administration.

Meanwhile, the Pollution and Asthma Risk: an Infant Study (PARIS) birth cohort study, which included 788 children, found that higher prenatal exposure to NO, particularly during the second trimester, was linked to decreased mid-expiratory flow at ages 8 to 9 years. Although no significant associations were found across the entire cohort for other lung function measures, both postnatal and lifetime exposure to traffic-related air pollution were associated with reduced forced expiratory volume in 1 second and forced vital capacity in children who experienced early repeated lower respiratory infections or had allergic sensitization.^181^ Special attention should also be given to the impact of air pollution on the respiratory health of children living in urban and suburban industrial areas. The most critical periods of exposure, whether during prenatal, perinatal, early childhood, or more recent, are not yet fully understood. Although there are few longitudinal studies available, evidence indicates that children exposed to air pollution from birth are at greater risk of developing chronic respiratory conditions.^182^,^183^ Research indicates that exposure to PM_2.5_, primarily linked to traffic-related air pollution, along with secondary sulfate sources associated mainly with industrial activity, significantly worsens respiratory symptoms in children.^184^ **Figure 4** illustrates the effects of air pollution during pregnancy on maternal and child health across the developmental stage.

**Figure 4:**
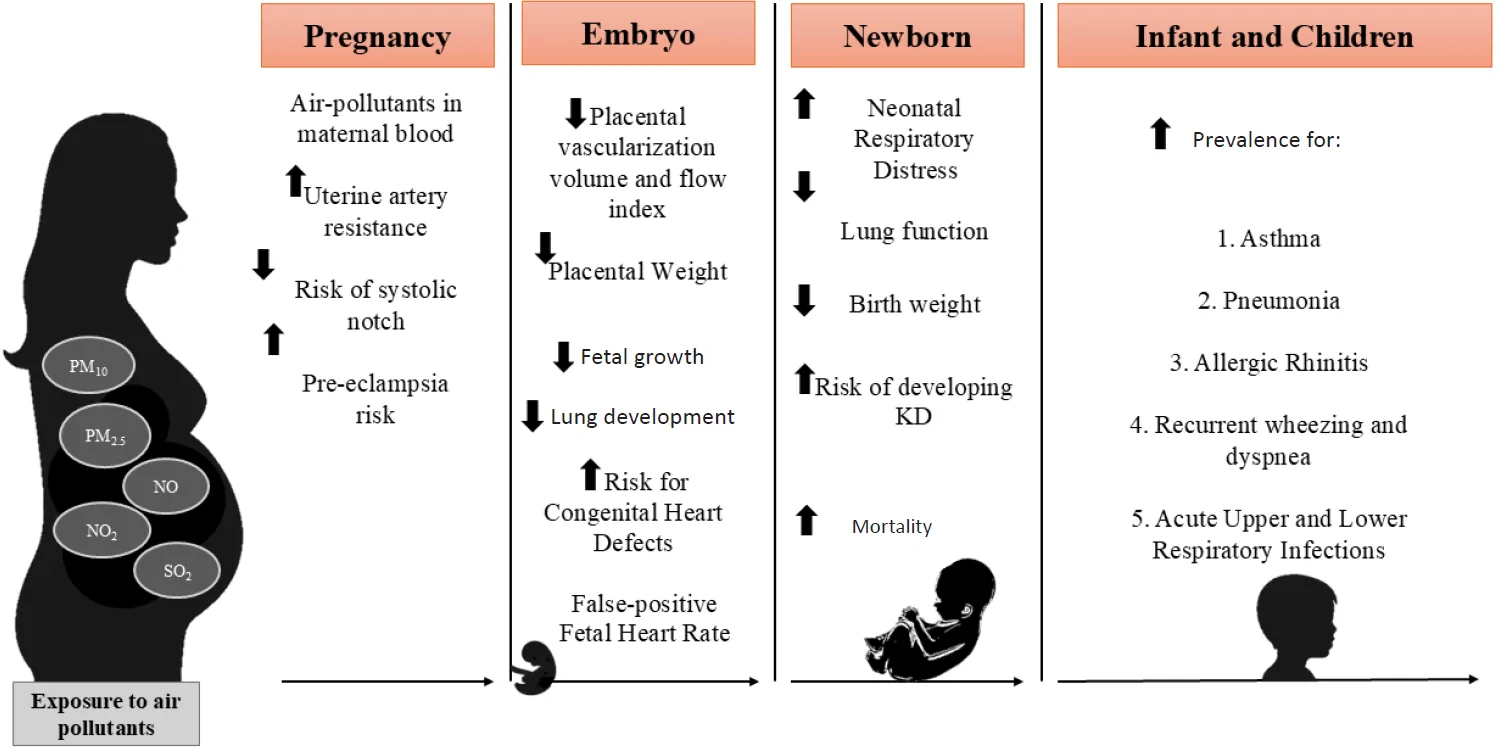
Effects of air pollution during pregnancy on maternal and child health across developmental stages. Air pollution during pregnancy adversely impacts maternal health, fetal development, and increases the risk of respiratory and systemic diseases in newborns, infants, and children. Created with Microsoft PowerPoint and Adobe Photoshop. ↑: Increase; ↓: decrease; KD: Kawasaki disease; NO_2_: nitrogen dioxide; O_3_: ozone; PM: particular matter; PM_10_: coarse PM (mean aerodynamic diameter 10–2.5 μm); PM_2.5_: fine PM (mean aerodynamic diameter 2.5–0.1 μm); SO_2_: sulfur dioxide; NO: nitric oxide.

### Studies in patients with cardiovascular disease, heart failure and circadian syndrome

Evidence shows that HF-related hospitalizations increase significantly with air pollution exposure, and this rise is greater than that observed for other chronic diseases. This supports a strong link between air pollution and both the exacerbation of HF symptoms and heightened vulnerability among individuals living with HF.^185^ Yang et al.^186^ by performing an updated systematic review and meta-analysis, reported that CO had the strongest link to HF events (3.46% increase per 1 ppm), followed by SO_2_ (2.20%) and NO_2_ (2.07%) per 10 ppb. O_3_ was also positively associated (0.95% per 10 ppb). PM_2.5_ and PM_10_ were both significantly linked to HF, with 1.29% and 1.30% increases in hospitalization or death per 10 μg/m^3^, respectively.

In a large UK Biobank cohort of 48,850 individuals with circadian syndrome, long-term exposure to various air pollutants, including PM_2.5_, PM_10_, PM_2.5_ absorbance, NO_2_, NOx, and an overall air pollution score, was significantly associated with major CVD subtypes.^50^ Interestingly, PM_2.5_ was linked to increased risks of coronary heart disease, HF, and stroke, PM_2.5_ absorbance was associated with HF, NO_2_, NO_x_, and the air pollution score were associated with increased risk of coronary heart disease, atrial fibrillation, HF, and stroke. Over a 12-year follow-up, 12,570 CVD cases were documented, while multistate regression models revealed that per-interquartile range increases in pollutant levels were linked to elevated hazards for transitions from circadian syndrome to CVD and from CVD to death.

### Studies in patients with cardiopulmonary or pulmonary diseases

Air pollution has been significantly associated with COPD exacerbation and mortality. In a longitudinal study, Kariisa et al.^187^ found that long-term exposure to PM_2.5_ was associated with worsening symptoms and a more rapid decline in lung function over a 5-year period among 1218 patients with very severe COPD. Additionally, even short-term exposures in ambient PM_2.5_ and PM_10_ levels were linked to symptom exacerbation and decreased lung function, highlighting the vulnerability of this patient population to both chronic and acute air pollution exposure. Literature shows that acute declines in air quality significantly increase the risk of COPD exacerbations, with gas pollutants having the most immediate effects on the first day of exposure, while the impact of PM peaks around 3 days later.^188^ In China, Liang et al.^189^ found that hospitalizations due to COPD exacerbations increased on days with high PM_10_, PM_2.5_, and SO_2_ levels, particularly during the warm season and among older adults and women with COPD. Similarly, a U.S. Medicare study involving 11.5 million recipients showed that a 10 µg/m^3^ increase in PM_2.5_ was associated with a 2.6-fold increase in COPD-related hospitalizations.^190^ A recent study in Greece found that while exposure to PM_2.5_, NO_2_, and black carbon was associated with increased COPD mortality, the associations did not reach conventional statistical significance.^191^ However, a stronger and statistically significant association was observed when using the log-transformed NO_2_ values. In contrast, higher levels of O_3_ and greater greenness were significantly associated with lower COPD mortality. These findings suggest a potential adverse effect of air pollution (especially NO_2_) on COPD mortality, while greenness appears to have a protective role.

Moreover, air pollution has been associated with worse outcomes in patients with idiopathic pulmonary fibrosis (IPF) and fibrotic interstitial lung diseases. In fibrotic lung diseases, growing evidence suggests that specific air pollutants are associated with adverse clinical outcomes. Notably, PM_2.5_ has been linked to IPF exacerbations,^192^ increased mortality,^193^ greater disease severity,^194^ a higher prevalence of hypersensitivity pneumonitis,^195^ and the presence and progression of interstitial lung abnormalities.^196^ PM_10_ exposure has also been associated with IPF exacerbations,^192^ increased disease incidence,^197^ and progression as reflected by a decline in forced vital capacity.^198^ Similarly, NO_2_ has been implicated in IPF exacerbations,^192^ as well as in increased IPF incidence^197^ and greater disease severity.^194^ In contrast, O_3_ has so far only been associated with IPF exacerbations.^192^

But what about other long-term lung diseases, such as asthma? Does air pollution affect only asthmatic children or adults? In a Peruvian study of children and young adults aged 9–19 years, 67% of whom had moderate to severe asthma, higher exposures to PM_2.5_, black carbon, and NO_2_ over a 6–9-month follow-up were independently associated with poorer asthma outcomes.^199^ These included worse asthma control, reduced asthma-related quality of life, and increased healthcare utilization. Notably, each interquartile range increase in PM_2.5_ and NO_2_ was linked to 59% and 34% higher odds of uncontrolled asthma, respectively. In the Sax Institute’s 45 and Up Study conducted in Sydney, a region with relatively low air pollution, long-term exposure to low levels of PM_2.5_ and NO_2_ was positively (though not significantly) associated with hospital admissions for asthma in adults over 45 years old, indicating that even minimal pollutant exposure may impact asthma outcomes.^200^ Additionally, data from Hubei, China, showed that between 2013 and 2018, 4454 asthma-related deaths occurred across all age groups, with short-term exposure to PM_2.5_, NO_2_, and O_3_ significantly increasing mortality risk, while PM_10_, SO_2_, and CO had no significant association.^201^ **Table 2** summarizes the cardiovascular and pulmonary health effects associated with major air pollutants in patient and healthy populations, highlighting their impact on clinical outcomes.

**Table 2:** Overview of cardiopulmonary health outcomes linked to major air pollutants

Air pollutant	Cardiovascular outcome	Pulmonary outcome
PM_2.5_	↑ CVD risk	↑ COPD exacerbations
	↑ IHD	↑ Asthma mortality
	↑ HF risk	↑ Acute lower respiratory diseases mortality
	↑ HF incidence, hospitalization and mortality	↑ Asthma symptoms
	↑ Vascular calcification	↓ Asthma outcomes
	↑ Stroke incidence	↓ Lung function
	↑ Hypertension	↑ IPF progression
	↑ Circadian syndrome risk	↑ ILD severity
	↑ KD risk	
	↑ Pneumonia diagnosis	
	↑ LVF impairment	
PM_10_	↑ HF risk	↑ COPD exacerbation and hospitalization
	↑ LVF impairment	↑ IPF exacerbations
	↑ Stroke incidence	↓ Lung function
	↑ Mortality	↑ Asthma in CVD
	↑ Pneumonia diagnosis	
PM_1.0_	↑ Stroke events	↑ COPD symptoms
	↑ CVD mortality	
	↑ Pneumonia diagnosis	
	↑ Atrial fibrillation	
NO_2_	↑ Hypertension	↑ Asthma symptoms and mortality
	↑ Heart disease	↓ Asthma control
	↑ HF incidence, hospitalization and mortality	↑ COPD mortality
	↑ LVF impairment	↑ IPF severity and progression
NOx	↑ Heart disease	↑ IPF incidence and exacerbations,
	↑ Hypertension	↑ COPD exacerbations and mortality
SO_2_	↑ HF incidence, hospitalization and mortality	↑ COPD and asthma exacerbations
	↑ CVD mortality	
	↑ KD	
CO	↑ HF events	↑ Asthma hospitalizations
	↑ HF incidence, hospitalization and mortality	↑ COPD mortality (less consistent)
O_3_	↑ CVD risk	↑ COPD and asthma mortality
	↑ Hypertension	
	↑ Cardiopulmonary mortality (mixed evidence)	
Black carbon	↑ CVD mortality	↓ Asthma control
	↑ Possibly HF in long-term exposure	↓ Lung function in asthma and COPD patients

↑: Increase; ↓: decrease; CO: carbon monoxide; COPD: chronic obstructive pulmonary disease; CVD: cardiovascular disease; HF: heart failure; IHD: ischemic heart disease; ILD: interstitial lung disease; IPF: idiopathic pulmonary fibrosis; KD: Kawasaki disease; LVF: left ventricular function; NO_2_: nitrogen dioxide; NO_x_: nitrogen oxides; O_3_: ozone; PM: particular matter; PM_1.0_: ultrafine PM (mean aerodynamic diameter 1.0 μm or less); PM_10_: coarse PM (mean aerodynamic diameter 10–2.5 μm); PM_2.5_: fine PM (mean aerodynamic diameter 2.5–0.1 μm); SO_2_: sulfur dioxide.

## Air Pollution and COVID-19: Interactions and Health Benefits of Cleaner Air

During the COVID-19 pandemic, global lockdowns led to an unprecedented reduction in outdoor air pollution, with significant improvements in air quality observed in America and several Asian and European countries. In China’s Yangtze River Delta, PM_2.5_ levels dropped by 25.4% to 48.1%, although O_3_ levels paradoxically increased by 20.5% during the highest level of lockdown starting on 24 January 2020.^202^ In Delhi, India, PM_2.5_, PM_10_, and NO levels decreased by 40–60%, with similar trends observed across the country.^203^ South Korea also reported a 45.45% reduction in PM_2.5_ levels in March 2020 compared to March 2019, along with declines in PM_10_, NO_2_, and CO, while O_3_ levels remained unchanged.^203^ Greater reductions in pollution were noted in cities with higher pre-lockdown pollution levels, such as Wuhan, Mumbai, and Daegu, whereas cities with already low pollution levels showed minimal changes.^204^ In Western Europe and the U. S., a notable reduction in NO_2_ pollution, ranging from 20% to 38%, was observed during the lockdown period. European cities, such as Madrid, Barcelona, and Milan, saw declines between 30% and 40% compared to the same period in 2019.^205^ Additionally, satellite data revealed a 30% decrease in the NO_2_ column over North America and Europe during the spring season of COVID-19 pandemic period.^206^ While these short-term improvements demonstrate the potential effectiveness of large-scale emission reductions, they were largely temporary, as pollutant levels rebounded once restrictions were lifted. This shows the necessity for sustainable and structural mitigation strategies, such as investment in cleaner transportation systems, industrial transformation, and the expansion of renewable energy, rather than reliance on temporary or disruptive interventions such as lockdowns.

Based on the above, it can be concluded that the COVID-19 lockdown created a unique opportunity to investigate the health effects of improved air quality. Despite growing evidence on the harms of air pollution, studies examining the health benefits of reduced exposure, particularly in relation to cardiovascular and respiratory outcomes in both healthy populations and individuals with chronic heart or lung diseases, remain scarce. Nevertheless, a limited number of studies have examined the impact of low ambient air pollution levels in vulnerable populations such as post-stroke patients, asthmatic children, lung cancer patients, and in the context of birth outcomes, including infant birth weight.

Early studies reported a notable decline in stroke admissions worldwide during the initial peak of the pandemic, with the World Stroke Organization confirming these results. While this drop may partly reflect patients avoiding medical care due to fear or restricted access to emergency services, it was also possible that lower air pollution contributed to a reduced incidence of stroke.^207^,^208^ Hameed et al.^209^ reported a decline in stroke admissions during the COVID-19 pandemic at a major tertiary care hospital in Karachi, Pakistan, alongside a notable improvement in air quality, as indicated by reduced Air Quality Index (AQI) levels, suggesting that the drop in AQI was attributed to strict lockdown measures implemented to curb the spread of the virus.

Furthermore, given the limited real-time data on environmental exposure and lung function in asthmatic children during the COVID-19 pandemic, the study by Lin et al.^210^ showed that levels of ambient air pollutants, particularly NO_2_, CO, and PM_10_, were significantly reduced during the lockdown compared to pre- and post-lockdown periods. Using real-time smartphone-based monitoring, they found that NO_2_ and SO_2_ were consistently associated with reduced peak expiratory flow in asthmatic children across multiple lag days. CO exposure was linked to decreased peak expiratory flow only in mite-sensitized children, highlighting the role of allergen sensitivity in modulating the effects of pollution. Seasonal variation was also evident, with spring showing stronger associations between pollutant exposure and reduced lung function.

Similarly, Aix et al.^211^ examined the impact of COVID-19 lockdowns on air quality and associated health outcomes in the Grenoble area, comparing data from 2020 to a reference period (2015–2019). During the lockdowns, significant reductions were observed in NO_2_ (–32%), PM_2.5_ (–22%), and PM_10_ (–15%) levels, while O_3_ increased by 10.6%. The most substantial health benefits were linked to declines in PM_2.5_ and NO_2_. Short-term risk reductions included a 3.2% decrease in pediatric asthma emergency visits and a 1.5% decrease in respiratory-related hospitalizations. Long-term benefits included reductions in low birth weight (–8%), overall mortality (–3.3%), and lung cancer (–2%) related to PM_2.5_, and a 0.96% drop in mortality related to NO_2_.

## Updates in Air Quality Standards: International Variations and Links to Cardiorespiratory Health Outcomes

### Air quality standards

Based on the U.S. EPA report for 2020,^212^ air quality standards vary by pollutant and exposure duration. For PM_2.5_, the short-term standard was 35 μg/m^3^ over 24 hours, while the long-term annual standard was 12.0 μg/m^3^. PM_10_ has a short-term standard of 150 μg/m^3^ over 24 hours, but no long-term standard was specified. SO_2_ was regulated with a short-term standard of 75 ppb over 1 hour, and there was no long-term standard listed. NO_2_ had both short- and long-term standards: 100 ppb over 1 hour and 53 ppb annually, respectively. O_3_ was regulated with a short-term standard of 0.07 ppm over 8 hours, but no long-term standard was defined. These standards were part of the National Ambient Air Quality Standards (NAAQS) for 2016 and were designed to protect public health by limiting exposure to harmful levels of air pollutants over different timeframes.^213^

More recently, the current NAAQS established under the Clean Air Act and enforced by the EPA set regulatory limits for six major air pollutants [PMs (PM_2.5_ and PM_10_), SO_2_, NO_2_, CO, lead (Pb) and O_3_] to protect both public health and the environment. These standards include primary limits, aimed at safeguarding sensitive populations such as children, the elderly, and individuals with respiratory illnesses, and secondary limits, which address broader environmental concerns like visibility and ecological damage. Each pollutant has specific averaging times (ranging from 1 hour to 3 months), concentration limits (measured in ppm, ppb, or µg/m^3^), and compliance forms based on exceedance frequency or multi-year averages. To be more precise, CO has primary standards of 9 ppm (8-hour) and 35 ppm (1-hour), not to be exceeded more than once per year. Lead has a rolling 3-month average limit of 0.15 µg/m^3^. NO_2_ has a 1-hour standard of 100 ppb and an annual mean of 53 ppb. O_3_ is limited to 0.070 ppm over 8 hours, based on the annual fourth-highest daily maximum concentration over three years. PM_2.5_ has both primary (12.0 µg/m^3^) and secondary (15.0 µg/m^3^) annual standards, and a 24-hour limit of 35 µg/m^3^, while PM_10_ has a 24-hour limit of 150 µg/m^3^, not to be exceeded more than once per year over 3 years. Lastly, SO_2_ has a 1-hour primary standard of 75 ppb and a 1-year secondary standard of 10 ppb. The EPA periodically reviews and updates these standards to reflect current scientific knowledge, although previously established limits may still apply in certain areas undergoing regulatory transition.^214^

### Tightening the limits of PM_2.5_ exposure

There are clear differences in national guidelines for permissible pollutant levels. For example, the recommended annual limit for PM_2.5_ varies significantly. Until recently, the WHO advised less than 10 µg/m^3^, the USA sets it at under 12 µg/m^3^, the European Union at 25 µg/m^3^, and China at 35 µg/m^3^.^215^ Reflecting growing evidence of harm at lower PM_2.5_ exposure levels, the WHO reduced its recommended annual mean PM_2.5_ guideline from 10 μg/m^3^ to 5 μg/m^3^ in 2021,^163^ and in 2023 released the sixth edition of its Ambient Air Quality Database, reporting annual mean concentrations of NO_2_, PM_10_ and PM_2.5_ across more than 8600 cities in over 120 countries.^216^ However, global PM_2.5_ levels vary widely, ranging from under 10 μg/m^3^ in countries like Canada to over 100 μg/m^3^ in South and East Asia, with a population-weighted global average of 46 μg/m^3^.^217^,^218^ Long-term exposure, even at low levels (e.g., 4.5 μg/m^3^), has been linked to increased respiratory and all-cause mortality, particularly in women, as shown in an Australian cohort study.^219^ In Canada, where average residential PM_2.5_ levels are under 10 μg/m^3^, studies have nonetheless identified associations between low-level exposure and increased overall mortality.^220^ One large study involving over 7.1 million Canadians found significant mortality risks even below the WHO’s strictest guideline of 5 μg/m^3^.^221^ Early-life exposure to air pollutants can also impair lung function into adolescence.^222^ With urbanization, climate change, and wildfires contributing to elevated PM_2.5_ levels, many cities are likely to exceed recommended thresholds, worsening health burdens.^223^

In February 2024, the U.S. EPA introduced updated AQI criteria for PM_2.5_, following a revision to the NAAQS that lowered the annual PM_2.5_ target to 9 µg/m^3^. The revised AQI thresholds reflect stricter upper concentration limits for several AQI categories. Specifically, the “Unhealthy for sensitive groups” category now corresponds to PM_2.5_ levels of 35.5–55.4 µg/m^3^ (AQI 101–150), while the “Unhealthy” category includes concentrations of 55.5–125.4 µg/m^3^ (AQI 151–200). The “Very unhealthy” category ranges from 125.5–225.4 µg/m^3^ (AQI 201–300), and the “Hazardous” level includes PM_2.5_ concentrations exceeding 225.5 µg/m^3^ (AQI 301+).^224^ Recommended actions escalate with AQI severity, ranging from no restrictions to avoidance of all physical activity outdoors. Corresponding estimated reductions in population mean exposure are 15%, 25%, and 35% for the Unhealthy, Very Unhealthy, and Hazardous categories, respectively^225^,^226^ (**Figure 5**).

**Figure 5:**
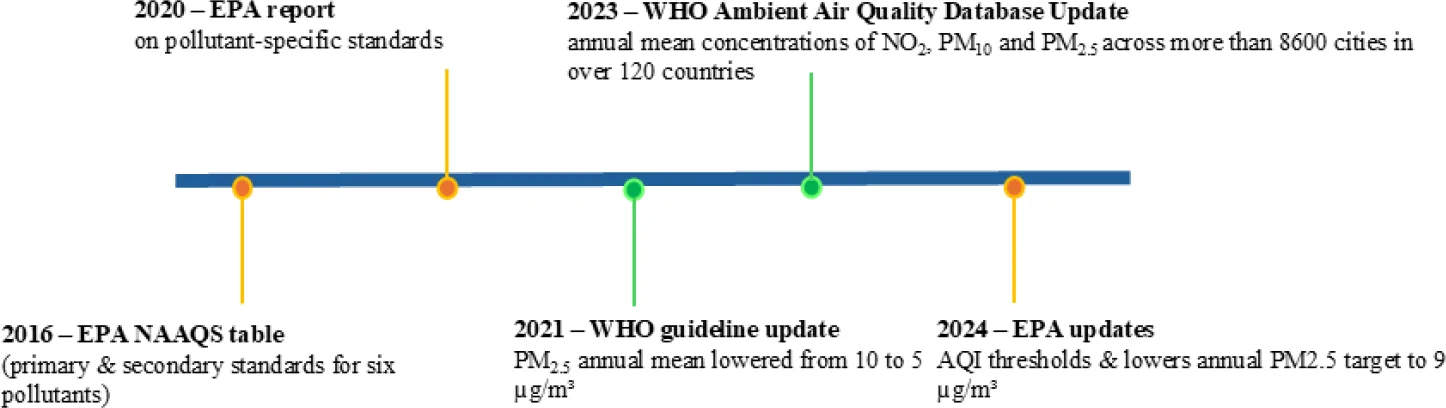
Timeline of air quality standards and guideline updates (2016–2024). Key milestones in international air quality regulation and guidance are shown, including EPA National Ambient Air Quality Standards (2016, 2020, 2024) and WHO updates (2021 guideline revision for PM_2.5_ and 2023 Ambient Air Quality Database update on NO_2_, PM_10_ and PM_2.5_ across > 8600 cities in > 120 countries). Created with Microsoft PowerPoint and Adobe Photoshop. AQI: Air Quality Index; EPA: U.S. Environmental Protection Agency; NAAQS: National Ambient Air Quality Standards; NO_2_: nitrogen dioxide; PM: particular matter; PM_10_: coarse PM (mean aerodynamic diameter 10–2.5 μm); PM_2.5_: fine PM (mean aerodynamic diameter 2.5–0.1 μm); WHO: World Health Organization.

Of note, the majority of deaths attributed to PM_2.5_ exposure are linked to CVD.^227^ While global pollution management has gradually improved, emerging evidence suggests that even low-level exposures (as low as 4.1–6.2 μg/m^3^) are associated with increases in non-accidental mortality.^228^ More critically, PM_2.5_ concentrations below 10 μg/m^3^ have still been shown to elevate cardiovascular mortality.^7^ These findings show the urgent need to assess the early health impacts of exposure to low-level PM_2.5_. Importantly, current evidence indicates that no level of air pollution can be considered entirely “safe.” Numerous high-quality studies have demonstrated that even exposure to PM_2.5_ concentrations below the most stringent standards remains associated with adverse health effects.^19^

## Mitigation Strategies and Future Directions

### Mitigation strategies

Several mitigation strategies have been explored to reduce air pollution exposure and its CVD impacts. The use of AQI warning systems has shown modest benefit in some populations. For example, the implementation of text-based alerts using an air quality warning system in Korea was initially associated with a reduced incidence of CVD (relative risk, 0.66), although the long-term benefits were unclear, and the effects were minimal among elderly or disabled individuals.^229^ Similarly, a Hong Kong-based system showed short-term reductions in hypertensive emergencies and myocardial infarction (relative risk 0.84 and 0.85, respectively), but no sustained benefit on overall CVD admissions.^230^ More recently, in a pioneering study by Brook et al.^225^ aimed to assess for the first time the effectiveness of current U.S. AQI activity guidelines in preventing atherosclerotic CVD (ASCVD) events among adults and explored the benefits of tailoring these recommendations based on individual ASCVD risk. Researchers calculated the number needed to treat (NNT) to prevent one ASCVD event per day across a range of 10-year ASCVD risk levels. They found that NNTs were extremely high, often exceeding millions, especially on days with AQI levels below 151, which account for the majority of days with elevated air pollution. Only under “unhealthy” air conditions (AQI 151–200) and among those at the highest ASCVD risk (18–20%) did current guidance (15% exposure reduction) achieve NNTs below 1 million. However, by tailoring recommendations to individuals with ASCVD risk ≥ 7.5% or ≥ 10% and applying stricter exposure reductions (35–50%), more favorable NNTs could be achieved. The study concludes that ASCVD risk is a key determinant of the potential benefit of AQI guidance and that individualized, risk-based activity recommendations may enhance the public health impact of air quality policies. However, in a Toronto-based study (2003–2012), air quality alerts were associated with a 25% reduction in asthma-related emergency visits but showed no significant impact on CVD or other cardiorespiratory outcomes. These results suggest that alerts alone have limited public health benefits and highlight the need for stronger, enforced measures to reduce air pollution.^231^ Increasing awareness through clinician-patient communication remains underutilized, with only 41% of health professionals discussing air quality, and just 16% doing so with CVD patients.^232^ Wearing masks, particularly N95 respirators, may reduce exposure to PMs, with modeling studies showing up to 39% reduction in respiratory hospitalizations,^233^ although real-world cardiovascular benefits are limited by poor mask fit and adherence.^234^,^235^,^236^,^237^,^238^,^239^ Portable air cleaners have shown promise in reducing PM and inflammatory biomarkers such as C-reactive protein (−7%) and IL-6 (−13%)^240^ and may lower systolic blood pressure by approximately 2.35 mmHg and diastolic blood pressure by 0.81 mmHg.^241^

Air conditioning can also lower indoor PM levels and reduce inflammation markers.^242^,^243^,^244^,^245^ For instance, Janssen et al.^245^ showed that cities with higher use of central air conditioning had weaker associations between PM_10_ pollution and hospital admissions for CVD and COPD. While Lin et al.^242^ found that indoor air pollution negatively affects cardiovascular health, increasing inflammation, oxidative stress, and reducing heart rate variability. These effects were strongest with open windows but were not seen when air conditioning was used, suggesting that air conditioning use can improve indoor air quality and protect cardiovascular health.^242^ Moreover, the use of cabin air filters (especially those with activated charcoal or high-efficiency designs) in vehicles has been linked with a significant reduction in pollutant levels and improvements in heart rate variability and blood pressure.^246^,^247^,^248^,^249^,^250^,^251^ Lastly, although policy-level interventions like diesel exhaust particle traps show cardiovascular promise,^252^ personal behavioral strategies remain crucial.

In addition to these mitigation strategies, studies have also shown that several dietary and lifestyle strategies may help moderate the cardiovascular effects of air pollution. Omega-3 fatty acids, particularly eicosapentaenoic acid and docosahexaenoic acid found in fish oil, have been shown to reduce inflammation and oxidative stress caused by PM_2.5_ exposure.^253^,^254^,^255^,^256^,^257^ Chen et al.^253^ noticed that short-term exposure to low levels of PM_2.5_ and O_3_ was linked to adverse cardiovascular responses in healthy adults with low omega-3 intake. These responses included changes in blood lipids, vascular inflammation, coagulation markers, and heart rate variability. However, these effects were not seen in individuals with a high dietary intake of omega-3 polyunsaturated fatty acids. This suggests that regularly consuming omega-3 fatty acids may provide cardiovascular protection, even when pollutant levels are below current regulatory standards.

Cocoa consumption may also offer vascular protection, with some evidence of reduced PM-induced vasoconstriction in children,^258^ although air pollution-specific data remain limited. Adherence to the Mediterranean diet appears to reduce CVD mortality linked to PM_2.5_ and NO_2_ exposure, with vegetables, whole grains, and monounsaturated fats providing the most protection.^259^ Furthermore, engaging in physical activity is generally beneficial for cardiovascular health. Research suggests that the benefits of exercise outweigh the risks associated with air pollution when outdoor activity is limited to 60 minutes or less in urban environments.^260^,^261^,^262^ However, in vulnerable populations or areas with high levels of PM_2.5_, it is important to exercise caution, as air pollution can reduce or negate the cardiovascular benefits of physical activity.^263^,^264^,^265^

### Future directions

To more effectively mitigate the cardiovascular and respiratory health impacts of air pollution, future studies should focus on developing integrated, personalized intervention strategies that target specific populations. Research should investigate how tailoring exposure reduction efforts to individual cardiovascular and pulmonary risk profiles, such as hypertension, HF, asthma, or COPD, can improve health outcomes. Rather than relying on broad public alerts, future interventions should incorporate dynamic, real-time responses such as individualized exposure warnings, adaptive physical activity recommendations, or automated indoor air quality controls. Longitudinal studies are needed to evaluate whether combining environmental measures (e.g., advanced filtration, ventilation, or cleaner transportation options) with supportive lifestyle interventions (e.g., anti-inflammatory diets and targeted physical activity regimens) can produce meaningful reductions in clinical cardiovascular and respiratory events. Special attention should also be given to high-risk populations, including older adults, people with chronic diseases, and socioeconomically disadvantaged groups, who may be more vulnerable yet less able to adopt protective behaviors. Ultimately, future research should aim to bridge the gap between environmental exposure science and patient-centered prevention strategies for long-term cardiopulmonary health.

In addition to the strategies mentioned above, it is essential to highlight the role of regulatory efforts such as the 2023 National Emissions Inventory (NEI) Documentation. This document outlines the U.S. EPA’s framework for compiling comprehensive air emissions data in collaboration with state, local, and tribal agencies. The guidance provided aligns with the Air Emissions Reporting Rule, ensuring consistency and accuracy in tracking emissions sources, a critical foundation for informed policy-making and targeted mitigation of air pollution’s cardiovascular and respiratory health impacts. It provides the best practices, timelines, and updated processes to ensure accurate, non-duplicative reporting of point, nonpoint, and mobile source emissions (excluding California, USA). Building on the successful 2020 NEI cycle, the 2023 plan includes features such as a single NEI version, iterative quality assurance reports, and expanded use of tools like the Wagon Wheel input templates and the Emissions Inventory System. Key updates include increased flexibility in nonpoint data submissions, revised source classification codes for Residential Wood Combustion, updated emission factors, and the inclusion of previously untracked sources, such as food trucks, asphalt roofing, and structure fires. Regional EPA offices are expected to play a supporting role in data collection and coordination. The final public release of the 2023 NEI is scheduled for March 31, 2026, with staggered deadlines for data submission throughout 2024 and early 2025. Accurate and comprehensive emissions inventories, such as the NEI, are critical tools for advancing our understanding of pollutant exposures that contribute to cardiovascular and respiratory disease burdens. These inventories support the development of targeted public health interventions and evidence-based regulatory policies by providing detailed, region-specific data on pollutant sources and concentrations. Future research should prioritize integrating high-resolution emissions data with health surveillance systems to more precisely model exposure-response relationships. Importantly, assessing the health impact of air pollution, particularly on cardiovascular and respiratory outcomes, requires the application of dose-response functions that are context-specific, considering local pollutant compositions, exposure durations, and population-level susceptibilities.^266^ This tailored approach is essential for producing accurate health risk estimates and informing both mitigation strategies and environmental health policies that reflect real-world exposure conditions.

Furthermore, future investigations should more deeply consider the multicomponent nature of PM_2.5_ and the cooperative interactions among its chemical constituents. Recent theoretical studies have demonstrated that heterogeneous mixtures of organics and inorganics can exhibit hierarchical synergistic effects during the formation of secondary organic aerosols, leading to distinct thermodynamic and structural properties that cannot be predicted by single-component analyses. For example, molecular dynamics simulations have shown that toluene-derived secondary organic aerosols often forms core–shell architectures stabilized by strong electrostatic interactions, with pyruvic acid acting as a molecular bridge that concentrates aromatic acids at the particle surface and reinforces hydrogen-bond networks. Such cooperative effects significantly lower the energy barrier for gas-to-particle conversion and shape nanoparticle morphology.^267^ Recognizing PM_2.5_ as a synergistic system of interacting constituents underscores the need for mechanistic studies that integrate chemical complexity into health risk assessments, thereby moving beyond reductionist single-component approaches.

To sum up, this review has both strengths and limitations. Air pollution remains one of the most pressing global public health challenges, contributing substantially to cardiovascular and respiratory morbidity and mortality. This review highlights not only the well-established mechanisms of injury but also emerging evidence on vulnerable populations, interactions with COVID-19, and the health impacts of recent policy-driven improvements in air quality. By integrating mechanistic insights, epidemiological findings, and international regulatory updates, our article provides a comprehensive and timely synthesis that distinguishes it from prior reviews. Importantly, we emphasize both the urgent need for stricter standards and the potential of individual- and policy-level mitigation strategies, offering a framework that may guide future research, policy, and clinical practice. However, this review has several limitations that should be acknowledged. First, it is a narrative rather than a systematic review, and therefore it may be subject to selection bias regarding the studies included. Second, cited literature is heterogeneous in terms of study design, populations examined, exposure assessment methods, and reported outcomes, which makes direct comparisons challenging. Third, most of the studies reviewed were published in English-language journals, raising the possibility of publication and language bias. In addition, the field of air pollution research is rapidly evolving, and new data may have emerged since the completion of this work. Finally, although we critically summarized current evidence, observational studies cannot fully exclude residual confounding, and causality cannot be established.

**Facts**

1. Air pollution is a leading global environmental health risk, responsible for millions of premature deaths annually, primarily from cardiovascular and respiratory causes.
2. Vulnerable groups, including pregnant women, children, and patients with chronic cardiovascular or respiratory diseases, experience disproportionate health impacts.
3. Reductions in air pollution during coronavirus disease 2019 lockdowns were associated with measurable improvements in cardiopulmonary health, highlighting the benefits of cleaner air.
4. Recent updates in international air quality standards (e.g., WHO 2021 and EPA 2024) reflect growing recognition that adverse health effects occur even at low pollutant levels.

**Open Questions**

1. What are the lowest safe exposure thresholds for PM_2.5_ and other pollutants, and do they vary by population subgroup (e.g., age, sex, and comorbidities)?
2. What biological mechanisms explain the link between air pollution and systemic diseases beyond the heart and lungs?
3. Can air quality interventions significantly reduce morbidity and mortality in high-risk groups?
4. How can international disparities in air quality standards and enforcement be reduced to ensure equitable protection of global public health?

## Data Availability

*Not applicable.*
